# Emotional Intelligence, Innovative Work Behavior, and Cultural Intelligence Reflection on Innovation Performance in the Healthcare Industry

**DOI:** 10.3390/brainsci13071071

**Published:** 2023-07-13

**Authors:** Rima H. Binsaeed, Zahid Yousaf, Adriana Grigorescu, Elena Condrea, Abdelmohsen A. Nassani

**Affiliations:** 1 Department of Management, College of Business Administration, King Saud University, P.O. Box 71115, Riyadh 11587, Saudi Arabia; rbinsaeed@ksu.edu.sa (R.H.B.); nassani@ksu.edu.sa (A.A.N.); 2 Higher Education Department, Government College of Management Sciences, Mansehra 21300, Pakistan; 3 Department of Public Management, Faculty of Public Administration, National University of Political Studies and Public Administration, Expozitiei Boulevard 30A, 012104 Bucharest, Romania; 4 Academy of Romanian Scientists, Ilfov Street 3, 050094 Bucharest, Romania; 5 Department of Economics, Faculty of Economic Science, Ovidius University of Constanța, Mamaia Boulevard 124, 900527 Constanta, Romania; elena.condrea@univ-ovidius.ro

**Keywords:** emotional intelligence, innovation performance, innovative work behavior, cultural intelligence

## Abstract

Innovation requires creativity, risk-taking, and the ability to manage change effectively, all of which are closely linked to emotional intelligence. Individuals with high levels of emotional intelligence are more flexible, adaptable, and resilient in technological advancements and are better able to respond effectively to new challenges and opportunities. Thus, this study aims to recognize the significant role of emotional intelligence, along with the mediation of innovative work behavior (IWB) and the moderation role of cultural intelligence in the attainment of innovation performance. This is quantitative research and for data collection, a questionnaire was used in healthcare institutions. The result shows that emotional intelligence is an antecedent of innovation performance. The finding also proved that IWB mediates the linkage between emotional intelligence and innovation performance. In addition, the outcomes show that cultural intelligence strengthens the relationship between emotional intelligence and innovation performance. However, the current dynamic business world has created an urgency to understand the linkage between the employee’s emotional intelligence and employee innovative performance, particularly taking into consideration the mediation effect of IWB. Emotional intelligence and innovation are closely linked, and innovative work behavior connects this link in a stronger way. This study offered a unique framework for achieving innovation performance through the nexus of emotional intelligence, innovative work behavior, and cultural intelligence.

## 1. Introduction

Employee innovativeness is an emerging challenge for HR managers of healthcare institutions in the current digitalized world [1]. Emotional intelligence is the ability to recognize, understand, and manage one’s own emotions, as well as those of others, that guides toward the achievement of innovation performance [2]. Supporting efforts toward emotional intelligence enhances employees’ innovative performance [3]. Therefore, this research focuses on how employee environment perspectives and personal ability, i.e., emotional intelligence, are appropriate and opportune to improve innovation performance. Emotional intelligence is an individual’s strength used for accomplishing their ability for self-management, societal management, self-consciousness, and social consciousness [4,5]. In spite of its relevance and significance, in the existing literature, there are limited researchers investigating the relationship between emotional intelligence and innovative performance of frontline employees in the context of a business industry. Close inspection of the related literature body defines the number of the potential features which promote innovative performance, but antecedents of innovative performance remain under-researched [6,7]. Nonetheless, the concept of emotional intelligence is relatively new among healthcare institution business researchers [8].

IWB refers to the behaviors that individuals engage in to create, develop, and implement new ideas within their organizations [9]. However, although emotional intelligence offers various reasons to the workers to maintain their present position with performing IWB and promptly make diverse and innovative things that lead to innovative performance, it attaches employees to their organization, job, and community [10]. This creates the urgency to understand the linkage between the employee’s emotional intelligence and the employee’s innovative performance, particularly taking into account the mediation effect of IWB. An individual’s knowledge and cultural ability improve employee IWB through interactions with people of other cultures [11]. This diversity influenced the linkage between emotional intelligence and innovative performance. Business industries are one of the most varied industries in the world, with an extremely mixed labor force and customers. Therefore, the cross-cultural dealings between them require IWB for the attainment of innovation performance [12]. Accordingly, this study recognizes how IWB acts as a bridge between EI and IP links.

Cultural intelligence is the capability to adapt and function effectively in diverse cultural settings [13]. Emotional intelligence is the potential to recognize, know, and utilize emotional information in relation to one’s self which leads to superior and effective performance [8]. Employees, customers, and organizations all have become multicultural. Therefore, the importance of effective cross-cultural management in healthcare is increasingly acknowledged as it permits the appropriate promotion of competitive healthcare services [14]. Thus, in this study, we suggest that employees with high levels of CI and EI at the same time will display more innovative performance. Therefore, the research supposes the moderation role of CI between EI and innovative performance relationship.

Prior studies have explored the different determinants of innovation performance such as cooperation networks [15], HRM [16], and the adoption of digital technologies [17], etc., while some other studies have explored the relationships between different variables in organizational dynamics, such as the roles of cultural intelligence and innovative work behavior [18], the influence of cultural intelligence on knowledge sharing [19], and the impact of employee emotional intelligence on enterprise innovation performance [20], etc. To the best of our knowledge, no study has been carried out with this empirical model. Therefore, to fill this gap, the study has three main objectives. First of all, it tests the influence of emotional intelligence on the innovative performance of workers. Secondly, it determines IWB as a mediator, i.e., a descriptive means to be familiar with the mentioned relationship. Finally, it examines how cultural intelligence strengthens the relationship between emotional intelligence and an employee’s innovative performance. This research is one of the few studies in the field of business that investigates a comprehensive framework for innovative performance. Examination of the IWB as a mediator and cultural intelligence as a moderator adds to the distinctness and uniqueness of the current study.

## 2. Literature Review Regarding the Absorptive Capacity Theory

The absorptive capacity theory was presented by Cohen and Levinthal in 1990. This theory suggests that a firm’s ability to recognize, assimilate, and apply new knowledge for research and development practices is crucial for innovation performance [21]. Absorptive capacity mainly emphasizes the competencies such as emotional intelligence, IWB, and CI that certain organizations improve so as to identify the significance of innovative knowledge [22]. Emotional intelligence is the aptitude to perceive, understand, and manage one’s own and others’ emotions, which can help individuals and organizations to learn and adapt to new information [2]. In the context of absorptive capacity theory, this study extends the boundaries of the prior literature that emotional intelligence can play an independent role in enhancing a firm’s ability to recognize and assimilate new knowledge. This study further explores that an individual with higher emotional intelligence may be better able to manage the emotions associated with change and uncertainty, which can facilitate the learning process. Innovation performance is likely to be mediated by an individual’s willingness to apply new knowledge, also known as IWB [9]. This study enriches the absorptive capacity theory on the role of culture intelligence and enlightens the moderating role of cultural intelligence between emotional intelligence and IWB that supports the understanding of the complex social and cultural norms associated with implementing new ideas, which could enhance innovation performance. Overall, this study suggests that emotional intelligence can facilitate in the development of absorptive capacity, which can in turn enhance IWB and ultimately lead to better innovation performance. However, the relationship between EI and IWB may be influenced by an individual’s level of cultural intelligence.

### 2.1. Emotional Intelligence

Emotional intelligence is the potential to recognize, know, and utilize emotional information in relation to oneself that causes superior and effective performance [3]. It is a useful list of personal qualities, along with cognitive intelligence that strongly aids in adaptation [4]. The key concept of emotional intelligence is the capability to think about one’s and others’ emotions, distinguishing and using the information to direct one’s and others’ actions and thoughts [10]. It is a complex variety of cognitive and perceptual intelligence abilities that are the determinant of the positive team member’s performance and the organizational outcome [18]. Emotional intelligence contains four skills, i.e., the capability to appeal to emotions for the cognitive facility, skills to identify emotional information, the ability to know oneself and others’ emotions, and the ability to gauge potential emotions [19].

### 2.2. Innovative Work Behavior

IWB is the actions of the employees directed at the application, generation, and implementation of new ideas, processes, products, and techniques to her/his job position, organization, and departmental unit. It includes searching for novel technologies, suggesting new plans for goal achievement, acquiring resources, and obtaining support for the implementation of new ideas [9].

### 2.3. Cultural Intelligence

Cultural intelligence refers to an individual’s ability to adapt successfully to different cultural settings. It is their capability to effectively relate to different cultural communities [12]. It is the ability of an individual to effectively, quickly, and comfortably perform in a dissimilar cultural environment [13]. Cultural intelligence consists of the four elements of cognitive ability, meta-cognitive processing, inspiration, and behavior. Cognitive ability focuses on existing information and knowledge of the customs, norms, and behaviors in diverse cultures. Meta-cognitive processing is the skill of dealing with the information that comes after experiencing different cultures. Cognitive motivation is derived from cultural incentives and experiences, i.e., the gained knowledge of different cultural varieties. Behavioral cognition is the capability to perform the appropriate nonverbal and verbal actions in a diverse cultural environment [23].

### 2.4. Innovative Performance

Innovative performance refers to creativity and the ideas used to improve and advance the procedures that boost the usefulness, performance, and significance of the services and products [1]. It is the ability to generate new behaviors, concepts, designs, procedures, and novel ideas for making inventive things and modernizing old concepts to be unique and novel concepts to act on job tasks [17]. Innovative performance is the arrangement of unique ways, novel practical policies, and new working ideas [19].

### 2.5. Emotional Intelligence and Innovative Performance

Emotional intelligence is connected to work performance, an employee’s behaviors, team performance, organizational commitment, turnover retention, and job satisfaction [2]. Emotional intelligence plays a key role in affecting employees’ readiness for complex and important dimensions of the learning, work, emotional, social, and intuitive processes, and creating novel ideas and products that lead to increased innovative performance [4]. Emotional intelligence influences individuals’ judgment and thinking processes by prompting a variety of information-processing strategies and new product development ideas. It helps in enhancing productivity and innovative performance in organizations [7]. Through possessing high emotional intelligence, employees have superior proficiency in cooperation and trouble- and conflict-solving ability, which is the foundation of innovation performance [10]. Moreover, employees with high levels of emotional intelligence have an improved perception of people’s sentiments and can handle their emotions [18]. When facing a job clash in the organization, emotionally intelligent individuals have the ability to direct actions and thinking that can successfully diffuse pressures and environmental demands. Therefore, these individuals tend to be more considerate of conflict in the organization and can recognize innovation strategies that lead to improved innovative performance [20]. Hence, emotionally intelligent employees can turn threats into opportunities, which leads to innovative performance. People with higher emotional intelligence manage conflicts in effective and appropriate ways and carry out good management behaviors to obtain better individual innovative performances [4]. Furthermore, employees with high emotional intelligence have a tendency to share effective suggestions and ideas to others, get aid from their colleagues and partners, and be familiar with how to sustain and maintain long-term supportive relations to improve innovative performance [20]. High Emotional Intelligence helps employees to articulate their own opinions and ideas properly, even if some conflict exists with others’ suggestions and opinions, in order to bring about a new innovative idea for increasing innovative performance [3]. Emotional intelligence in employees is concerned with job satisfaction, customer satisfaction, service performance, and organizational behavior and success [24]. Emotional intelligence is related to the voluntary behavior of individuals away from their main roles, helping employees to give creative ideas for promoting innovative performance [25,26]. Emotional intelligence enables employees to carefully handle their jobs and employ innovativeness in their work to create useful and novel ideas to obtain valuable innovative performance [10].

**Hypothesis** **1 (H1).** *Emotional intelligence predicts innovative performance.*

### 2.6. Innovative Work Behavior as a Mediator

Emotional intelligence builds an individual’s ability to make links such as social ties and personal attachments to improve their IWB, allowing them to provide unique ideas that enhance innovative performance [11]. Emotional intelligence is set as the base for higher IWB and this IWB motivates employees to be engaged in the generation of innovative ideas and subsequent behavior for increasing innovative performance in firms [9,27].

Emotional intelligence improves IWB dimensions, i.e., link, fit, and sacrifice. It is the individual’s perception regarding his fitness in the organization to acquire career objectives and goals in the available environmental infrastructure such as culture, climate, and comfort [9]. An individual’s link with the organization is the personal affection and sacrifice of giving up something valuable for the sake of others’ considerations, such as for co-workers, job stability, and career advancement [28]. These attributes of employees’ IWB built strong foundations for innovative performance. Hence, we argue that IWB is an outcome of emotional intelligence and is the driver of innovative performance, and it should act as a mediator between emotional intelligence and innovative performance.

Emotional empowerment enhances an individual’s attachment to their job and thus employees search for new methods to perform their activities [26]. It develops IWB so enthusiastically that an individual feels more attractive and delightful for attaining freedom. This perception of their secured future and freedom facilitates employees to behave, think, and perform innovatively which leads to overall innovative performance [27].

Emotional intelligence boosts employees’ enjoyment of performing various tasks together to improve their IWB in the terms of link, sacrifice, and fit to increase innovative performance in the firm [3]. IWB enhances an individual’s ability to design novel ideas and adapt new work attitudes which are prerequisites for innovative performance [14]. Emotional intelligence in employees makes sense of the self-confidence with which employees are capable of taking risks, such as novel idea generation and implementation [27].

Furthermore, emotional intelligence gives a detailed mechanism by which the IWB of the employees changes to bring about innovative performance [24]. Therefore, it is absolutely rational to theorize that IWB positively mediates between emotional intelligence and innovative performance. It is IWB that ensures the employees give sufficient time and due attention to the multiple tasks to be performed simultaneously, and that they can aptly adopt innovative performance [9]. If IWB acts as a mediation between emotional intelligence and innovative performance, then employees in this case will perform different tasks in more suitable ways [28]. It is IWB that reduces the chances of employee turnover in the business profession; therefore, IWB connects emotional intelligence with innovative performance [29]. Emotional intelligence consists of the three IWB tasks which are idea promotion, idea realization, and idea generation [26]. It demonstrates the generation and implementation of innovative novel ideas that deal with the enhancement of innovative performance and problems in the workplace [30].

**Hypothesis** **2 (H2).** *IWB mediates between emotional intelligence and innovative performance.*

### 2.7. Cultural Intelligence as a Moderator

Cultural intelligence refers to an individual’s capability to attract, comprehend, and explore multiple cultural emotional intelligence motives to do something accordingly in the multicultural state to promote innovative performance [31]. Cultural intelligence improves one’s own and others’ moods, personalities, and emotions, thus highlighting emotional intelligence’s effect and capacity to influence task and action performance, including innovative performance [32]. In this study, we suggest that employees with high levels of CI and EI at the same time will display more innovative performance. Therefore, the research supposes a moderation role of CI in the relationship between EI and innovative performance. For the support of improved emotional intelligence and innovative performance of employees, the influence of cultural intelligence has to be focused because these arrangements empower the employees to generate novel ideas and implement multiple tasks [33]. Cultural intelligence provides helpful informational swaps between employees and increases their emotional intelligence to support innovative ideas and feedback to management [34]. Employees with emotional intelligence have a preference for performing various jobs instead of giving attention to one specific activity [31]. Likewise, innovative performance also needs cultural intelligence to support employees to strengthen their efforts to make precious inputs concerning idea generation and idea realization [23]. Motivated employees create new design products, provide services, and increase their innovative performance in the firm [35]. Cultural intelligence has major influences on individual perception in organizations.

Similarly, cultural intelligence plays a moderating role between emotional intelligence and innovative performance. Furthermore, cultural intelligence builds up a stronger influence on the emotional intelligence and innovative performance of the employees by empowering their decision-making power. It advances top-down and bottom-up information streams through which managers can share emotional empowerment with their subordinates relating to organizational matters [36]. Such psychological IWB and moderation effects of cultural intelligence in terms of emotional intelligence are key factors that facilitate the handling of resources by staff members, along with making and implementing novel ideas to carry out new things. Alternatively, they lead to enhanced innovative performance [14,37]. Cultural intelligence is a regionalized decision-making process, it permits employees to do something independently and simply to attain innovative performance through their enhanced emotional intelligence behavior [12]. Moreover, we speculate that cultural intelligence encourages and motivates employees to take part in the decision-making process and has a stronger positive influence on emotional intelligence as well as increased innovative performance [38]. From the above arguments, we hypothesize that cultural intelligence positively moderates between emotional intelligence and innovative performance.

**Hypothesis** **3 (H3).** *Cultural intelligence acts as a moderator between emotional intelligence and innovative performance.*

**Figure 1:**
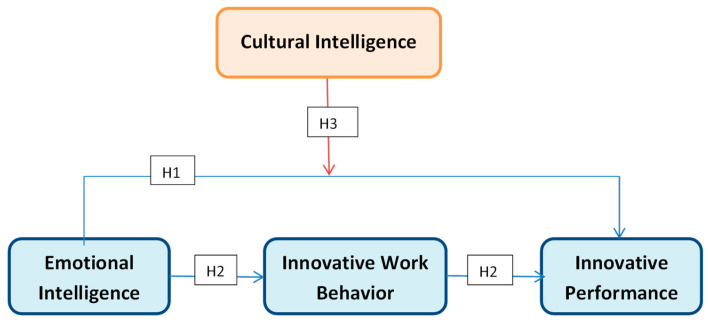
Theoretical framework.

In Figure 1 is presented the theoretical framework of the research.

## 3. Materials and Methods

This is a quantitative study and cross-sectional data have been gathered. This study was conducted in the healthcare industry. For the collection of data, 14 healthcare institutions from Saudi Arabia were selected and approached, and a list of 1400 employees and 70 managers and supervisors was collected from the HR databases of each healthcare institution. This data collection method was started through the help of five associate researchers who sent all questionnaires to the selected employees and managers through the HR supervisors of the healthcare institutions. The questionnaires contained a cover letter in the envelope which included the purpose and guidelines of the research, as well as the detailed variables with which to complete the questionnaire. Employees were asked to boost up their emotional intelligence and IWB. Managers, being direct controllers of the employees, were asked how they would improve the cultural intelligence and innovative performance of the employees. The associate researchers personally obtained the questionnaires from participants and directed them to complete every section of the questionnaire properly. The entire data collection process took three months and the final number of results from employees was 975. Out of these, only 775 were useable for analysis after corresponding with employees and managers regarding the survey’s outcomes. The concluding responses from managers showed that 55 out of 70 rated the cultural intelligence and innovative performance of their subordinate employees. During the process of data collection, the research associates followed all international and national standards, and members were informed about data confidentiality. To make sure that the scale could be generalized, we used pre-checked adapted items that had been previously employed by scholars. All items were rated on the five-point Likert scale, i.e., 1 = strongly disagree and 5 = strongly agree. To confirm the validity of the scales, we performed a pilot study to determine the accuracy of the questionnaires. All essential adjustments were done in terms of their design and wording. The demographic section of this questionnaire consists of the education, job status, age, and job experience of the employees and managers.

### 3.1. Measurements

#### 3.1.1. Emotional Intelligence

Emotional intelligence was examined through an eight-item scale which is adapted from the studies of [39,40]. A similar study was conducted by Khan et al. [41] on international students’ academic performance. The sample item is “Understand the emotions of others”.

#### 3.1.2. Innovative Performance

Previously, different research confided on the secondary and archival sources that collect data for the innovation contribution and output. However, in this research, we exploited self-reported survey questionnaires from managers, and directly sought information interrelated to innovative performance. This approach was used by author [42]. Compared with widely reported information, the self-reported approach has the obvious benefit of providing supplementary, reliable, and accurate data. IP questions “Evaluating different substitutes for the customer’s problem.” are based on the work of [42].

#### 3.1.3. Cultural Intelligence

The strengthening role of cultural intelligence in the relationship between emotional intelligence and innovative performance is reasonable on the basis of the research that speculates that adaptability to the culture improves an individual’s own and others’ emotions, personalities, and moods, thus accentuating emotional intelligence and its capacity to affect the performance of tasks and actions, which includes innovative performance. Cultural intelligence is measured with a 5-item scale adapted from [43].

#### 3.1.4. Innovative Work Behavior

IWB is measured by a 4-item scale that was previously used by [44,45]. IWB consists of three main measurements which are idea promotion, generation, and realization. A total of 4 questions were asked to measure this.

## 4. Results

### 4.1. Reliability and Validity

This study analysis was conducted using SPSS 18.0 and SEM. Table 1 demonstrates the outcomes of the convergent validity, AVE, and alpha. To test discriminant validity, the Fonell–Larcker [46] technique was used. The outcomes in Table 1 show satisfactory results for C.R., AVE, and alpha values.

### 4.2. Descriptive Statistics

Table 2 presents the results of descriptive statistics and the correlation of the construct utilized in this research. The results proposed that all the values are positively significant. There is a direct relationship between EI and IP as shown by the correlation coefficient value of 0.28, which is statistically important at a level of 1%. Likewise, the association between IWB and innovation performance is positive as revealed through coefficient values of 0.36, which is considerable at a level of 1%. Furthermore, cultural intelligence and IP are positively correlated with a coefficient value of 0.34.

According to Joerskog and Sorbom [47], the constructs’ validity was tested by Confirmatory Factor Analysis (CFA). In the current study, a 4-factor model was fitted to data and approved values of CFA were obtained (χ2 = 1045.42, df = 480; χ2/df = 2.178; RMSEA =0.05; CFI = 0.94; GFI = 0.93).

### 4.3. Hypothesis Testing

To decide whether to accept or reject this study hypothesis, we have utilized the SEM technique. Table 3 shows that emotional intelligence is positively predictive of innovative performance according to the results (β = 0.24 **, *p* < 0.001). Hence, H1 is accepted.

For testing the mediation of IWB between emotional intelligence and innovative performance, the analysis of Preacher and Hayes [48] was used. Table 4 illustrates the indirect influence of IWB between emotional intelligence and innovative performance (EI IWB IP). Path ‘a’ shows that EI predicts innovative performance (B = 0.348, t = 7.543, *p* = 0.000). Path ‘b’ shows the direct impact of IWB on innovative performance (B = 0.258, t = 7.526, *p* = 0.000). Path ‘c’ proposed the total effect of EI on IP (B = 0.244, t = 3.732, *p* = 0.000). Path ‘c’ showed that, when innovative work behavior was controlled, the direct effect of EI on IP was summarized and non-significant, showing a mediation outcome (B = 0.148, t = 1.1213, *p* = 0.128 Ns.). Path ‘ab’ presents outcomes of the indirect impact in the last ration of Table 4. Indirect impact outcomes show that IWB plays a mediation role (Beta = 0.148, Lower = 0.1240 to Upper = 0.2870. The normal assessment also led us to check the mediation impact of IWB. Hence, H2 was accepted and it was confirmed that the link between EI and IP is intermediated by IWB.

Table 5 shows the moderating result of CI on the direct association between EI and IP. The findings indicate that cultural intelligence performs an important role in the connection between EI and IP, i.e., (β = 0.22 **, *p* < 0.001).

These results confirm Hypothesis 3, showing that CI acts as a moderator between EI and IP. This is a consistent finding for entrepreneurs and management to stimulate the development of an organizational culture meant to stimulate the individuals’ cultural intelligence.

## 5. Discussion

The key objective of our study was to examine the linkage between emotional intelligence and innovative performance. Moreover, the mediating functions of IWB and the moderation effect of cultural intelligence have been also tested. Within this regard, we have tested three hypotheses. H1 of our study suggested that emotional intelligence positively affects innovative performance. Researchers also expect that emotional intelligence plays a key role in affecting employees’ readiness for the complex and important dimensions of the learning, work, emotional, social, and intuitive processes, and the creation of novel ideas and products that lead to increased innovative performance [4]. Emotional intelligence influences individuals’ judgment and thinking processes by prompting a variety of information-processing strategies and new product development ideas. It helps in enhancing productivity and innovative performance in organizations [7]. These outcomes confirmed H1 and showed that employees with emotional intelligence are in an improved position to develop, generate, and implement inventive ideas by performing different tasks together. The results of Hypothesis 2 disclose that IWB positively plays a mediating role between emotional intelligence and innovative performance. This is factual, particularly in the context of developing countries as employees have been performing various jobs simultaneously and also working in an inventive way when they were connected to their job. Scholars argue that emotional intelligence improves an individual’s ability to make links such as social ties and personal attachments to improve their IWB. In addition, they provide unique ideas that enhance innovative performance [11]. Emotional intelligence sets the base for higher IWB and this IWB motivates employees to be engaged in the generation of innovative ideas and subsequent behavior for increasing innovative performance in firms [9,27]. The outcomes confirm that IWB is determined through emotional intelligence and translates into improved innovative performance. The final hypothesis of this research investigated the moderating role of cultural intelligence and the extent to which it is capable of strengthening the dominant effect of emotional intelligence on innovative performance. The researchers hypothesize that cultural intelligence is a regionalized decision-making process, permitting employees to do something independently and simply attain innovative performance through their enhanced emotional intelligence behavior [12]. Moreover, we speculate that cultural intelligence encourages and motivates employees to take part in the decision-making process and strengthens the positive influence of emotional intelligence on increased innovative performance [38]. The end result showed that the emotional intelligence of the employees can improve their innovative performance through cultural intelligence in the organizations. Cultural intelligence plays an important key role in the achievement of desired goals. Hence, it is completely comprehensible that employees with high emotional intelligence want to complete multiple tasks simultaneously and seek various novel innovative ideas and ways. They perform different independent actions that are probably possible through cultural intelligence. In summary, while employees are directly involved in decision-making processes and problem-solving conversations, they will perform better to give their enhanced innovative performance. Therefore, the impact of the employee’s improved emotional intelligence on innovative performance will be highly speedy and effective when the moderating role of cultural intelligence is added.

### 5.1. Theoretical Implications

This study adds valuable inputs to the theory in many ways. Firstly, we discover exciting dimensions of the employee’s higher emotional intelligence in terms of innovative performance. Prior investigators point to various features which determine innovative performance, such as the mediation effect of IWB and the moderation effect of cultural intelligence. However, the present research is the first attempt to recognize that innovative performance could be begun through employees with higher emotional intelligence. The work of prior scholars only linked emotional intelligence with an employee’s task/job performance, sales growth, satisfaction, and income when evaluating organizational performance. As part of the historical flashback, the motivational perspective of functionalized and determined supportive activities of emotional intelligence can be helpful in determining innovative performance. Therefore, this study contributes to the existing literature by demonstrating that the emotional intelligence of employees can be related to innovative performance. Accordingly, instead of focusing on the direct linkage between an employee’s higher emotional intelligence and their innovative performance, we have studied the vital role of IWB in said relation. This study added to the theory that managers and supervisors psychologically motivate and encourage emotionally intelligent employees by involving them in decision-making. Moreover, managers’ admiration in terms of relational leadership also acts as a strong basis for the higher emotional intelligence of their employees and improves their attitude toward increasing their innovative performance. This research has extended the traditional method of thinking and combined the scattered work of the prior studies that believed that the emotional intelligence of the employees acts as a precursor for the employees to increase their innovative performance. The outcomes of our research also show the mediation effect of IWB between emotional intelligence and innovative performance in employees. The study results prove the mediation effect of IWB between emotional intelligence and innovative performance in employees. Moreover, our study’s purpose is to precede concepts of emotional intelligence in workers and remove the suspicion surrounding the irregularities and narrow extents of emotional intelligence in the given literature body. This study provides an understandable model to enlighten the results of emotional intelligence by boosting their scope. This research further questions the effect of emotional intelligence on employees and their innovative performance from a motivational viewpoint through which employees get engaged with their jobs. From the empirical evidence, we strongly suggest that the mediating role of IWB in linking emotional intelligence and innovative performance is the major input to the theory. This research also proposes that the impact of emotional intelligence on innovative performance is stronger when cultural intelligence is high. Our study results show how cultural intelligence positively plays a moderating role between emotional intelligence and innovative performance. This research not only discusses the outcomes of cultural intelligence, it also extends the existing body of literature by adding that cultural intelligence acts as a moderator for enhancing the impact of emotional intelligence on innovative performance. It is a universal concept that employees with higher emotional intelligence are interested in doing various jobs simultaneously, while elastic cultural intelligence facilitates employees to provide inventive concepts for performing multiple jobs at once.

### 5.2. Practical Implications

The employees of the organizations must perform multiple tasks at the same time in their regular activities. Therefore, they always need to search for innovative ideas to perform various jobs. Innovative performance is the generation of new inventive ideas that are helpful for administrative processes and improving services. The outcomes of this study contribute to the practical management of individuals such as managers and supervisors, as well as the working staff of the organizations. Firstly, managers need to give attention to their working staff and subordinates who perform several tasks at once and create proper working environments which enhance the innovative performance of the employees. This research confirms that employees with high levels of emotional intelligence increase their innovative performance, signifying that managers should emphasize the higher emotional intelligence ability of these employees to encourage them to increase their innovative performance. The outcomes of this research propose that management use different skills to maintain strong relations with its subordinates. These relational skills act as the base for the enhancement of the emotional intelligence of employees to increase their innovative performance. However, in practice, management concentrates on constant appreciation and relational management. This psychological empowerment can improve the emotional intelligence of the employees, consequently boosting their innovative performance. Furthermore, an employee’s improved emotional intelligence ultimately increases their innovative performance. Secondly, managers need to consider the importance of IWB and use this to boost the employee’s innovative performance. This study proposed that emotional intelligence has an indirect impact on the innovative performance of IWB. Therefore, a manager focuses on their subordinates to encourage and motivate them to be engaged in their jobs by improving relations through attractive packages, team building, etc., eventually strengthening their innovative performance. Finally, managers encourage cultural intelligence and valuable contributions from their working staff and subordinates. This research helps both managers and employees by considering the moderating role of cultural intelligence between the emotional intelligence of the employees and their innovative performance. Although emotional intelligence acts as a mechanism of innovative performance among employees, the addition of cultural intelligence speeds up the process.

Members of staff with the capability to “think and imagine out of the box” could make a competitive framework for firms and deal with multifaceted and complex confrontations and fast-changing settings in the modern world [4]. Accordingly, increasing competition from organizations in the business sector puts a superior emphasis on innovative performance to boost and enhance customers’ satisfaction and service quality [7].

Innovative performances direct toward generation of innovative and new ideas resulting into new service and the product development [49,50]. The novel services and the product developed by innovative ideas enable business firms to respond toward changing trends and needs of customers to stay highly competitive [51].

### 5.3. Limitations and Future Directions

This research has several limitations that should be considered in future studies. Firstly, the study focuses primarily on the individual-level factors that contribute to innovation performance and does not fully account for the broader organizational and contextual factors that may influence innovation outcomes. However, future research should consider incorporating a broader range of variables, such as organizational culture, leadership, and resources, to provide a more comprehensive understanding of the drivers of innovation performance. Secondly, this study used multiple sources of data, such as peer and supervisor ratings, to provide a more comprehensive assessment of these constructs. Therefore, future research should consider self-reported measures to explore more outcomes of these variables. Thirdly, this study is based on cross-sectional data, which limit the ability to make causal inferences about relationships between these variables. Hence, future research should incorporate qualitative and longitudinal designs to provide a more comprehensive understanding of these variables. Lastly, the study is limited by its sample; thus, future research should consider replicating these findings across a wider range of cultures and organizational contexts to provide a more comprehensive understanding of the generalizability of these relationships.

## 6. Conclusions

In summary, this study’s findings offer significant evidence that the ability to innovate is greatly influenced by emotional intelligence. Further research revealed that IWB serves as a mediator in the association between emotional intelligence and innovation performance, suggesting that people with higher emotional intelligence are more likely to engage in innovative behaviors, improving innovation outcomes. The results also show how crucial a role cultural intelligence plays as a moderator, showing that people with higher cultural intelligence are better able to use their emotional intelligence for improved innovation performance. These findings highlight the importance of emotional intelligence, IWB, and cultural intelligence in fostering innovation within organizations, demonstrating the need for organizations to prioritize the development of such competencies in staff members to drive effective innovation initiatives and attain competitive advantages.

## Figures and Tables

**Table 1:** Display of F.L., C.R., and AVE values.

	Items	F.L.	α	C.R.	AVE
Emotional Intelligence	8	0.75–0.78	0.84	0.91	0.74
Innovative Work Behaviour	4	0.73–0.85	0.85	0.92	0.76
Cultural Intelligence	5	0.77–0.83	0.82	0.95	0.72
Innovative Performance	4	0.75–0.86	0.86	0.94	0.73

Source: authors’ computation.

**Table 2:** Correlations.

Variable		Mean	SD	α	1	2	3	4	5	6	7	8
1	B.Size	2.04	0.03	0.83	1.00							
2	B.Age	1.24	1.45	0.85	0.123 **	1.00						
3	Edu.Res	3.63	0.31	0.83	0.111 **	0.742 *	1.00					
4	Exp.Res	2.65	1.58	0.88	−0.120	0.057	1.341	1.00				
5	Emotional Intelligence	1.72	0.46	0.82	−0.063	−0.094	0.420	−0.111	1.00			
6	IWB	1.42	0.58	0.86	0.019	−0.192	0.193 *	−0.221	0.184 **	1.00		
7	Cultural Intelligence	3.52	0.73	0.81	−0.022	−0.213	−0.011	0.085 *	0.327 **	0.327 **	1.00	
8	Innovative Performance	0.21	0.49	0.83	0.031	−0.121	−0.053	−0.120	0.280 **	0.369 **	0.345 **	1.00

Note: * *p* ≤ 0.001, ** *p* ≤ 0.05.

**Table 3:** The effect of Emotional Intelligence on Innovative Performance.

Model	Hypothesis Detail	β	F	T	Sig	Remarks
Model 1	Emotional Intelligence → IP	0.24	14.073	0.153	0.001	Supported

Source: authors’ computation.

**Table 4:** Indirect Effect.

Paths Detail			Models	β	*t*-Value	SE	Sig
(Path.a) → I.V to Mediation			EI → IWB	0.348	6.543	0.052	0.001
(Path.b) → Direct impact of the mediator on dependent variable			IWB → IP	0.258	5.526	0.042	0.001
(Path.c) → Total impact on I.V on D.V.			EI → IP	0.244	2.732	0.057	0.001
(Path c’) → Direct influence of the I.V on D.V.			EI → IP	0.148	7.213	0.056	0.128
Mode. summary for D.V.: R^2^ = 0.1422; F = 34.7321; P = 0.000							
**Bootstrap-Indirect Impact [ab path]**							
**Model Description**	**Data**	**Boot**	**Bias**	**SE**	**Lower**	**Upper**	**Sig**
EI → IWB → IP	0.148	0.18	0.003	0.44	0.1240	0.2870	0.0000

Source: authors’ computation.

**Table 5:** Moderating effect of CI.

IP						
Detail	β	*t*-Value	β	*t*-Value	Β	*t*-Value
**Step-1**						
B.Size	0.08	0.14	0.02	3.31	0.02	0.66
B.Age	0.07	0.21	0.26	0.56	0.14	0.75
Edu.Res	0.09	0.39	0.08	0.11	1.31	1.41
Exp.Res	0.13	0.35	0.10	0.93	0.01	0.12
**Step 2**						
EI			0.36 *	6.95	0.34 *	2.52
CI			0.26 *	4.44	0.42 *	5.56
**Step 3**						
EI × CI					0.22 **	1.24
F		5.18 **		18.54 *		15.57 *
R2		0.05		0.24		0.27
R2				0.21		0.01

Source: authors’ computation. Note: * *p* ≤ 0.001,** *p* ≤ 0.05.

## Data Availability

Data available on request.
